# Prejudice, segregation and immigration laws —integration of the robot into the laboratory society

**DOI:** 10.1155/S1463924694000155

**Published:** 1994

**Authors:** Norman E. Fraley, Jr.

**Affiliations:** Express Analytic 31M Woodcreek Drive Downers Grove Illinois 60M5 USA

## Abstract

This paper addresses some serious issues about personnel morale, fears and hopes associated with and attributed to the laboratory robot. The introduction of the laboratory robot into the laboratory is examined from a managerial perspective. Human-rights and robot-rights issues are identified and addressed. Real world examples of how the integration of two high through-put robots affected the routine of a major industrial food laboratory are discussed.


Journal of Automatic Chemistry, Vol. 16, No. 4 (July–August 1994), pp. 139–141

### Prejudice, segregation and immigration laws—integration of the robot into the laboratory society

**Norman E. Fraley, Jr.**

*Express Analytic, 3131 Woodcreek Drive, Downers Grove, Illinois 60515, USA*

*This paper addresses some serious issues about personnel morale, fears and hopes associated with and attributed to the laboratory robot. The introduction of the laboratory robot into the laboratory is examined from a managerial perspective. Human-rights and robot-rights issues are identified and addressed. Real world examples of how the integration of two high through-put robots affected the routine of a major industrial food laboratory are discussed.*

A large number of people are not afraid of progress just so long as no one changes anything. The integration of the robot is very much like other major changes that can occur within a business. This paper examines some of the effects that the addition of automation, laboratory robotics in particular, can have on an analytical laboratory.

Two men were examining the output of the computer in their department. Eventually, one of them remarked, 'Do you realize that it would take 400 men 250 years to make a mistake this big?' In light of the advancements that the computer industry has given to all areas of society, specifically within the analytical laboratory, we are in a situation where we can get more done but make much bigger mistakes faster than ever before. One of the primary topics we need to examine is the part laboratory robots play in not only the productivity and efficiency of the laboratory, but also as the newest member of the laboratory society.

Express Analytic is a analytical service laboratory serving the food industry. Its goal is to provide the fastest, most accurate and realiable analytical service in the laboratory industry. In 1989, the US government proposed the Nutrition Labeling and Education Act. Express Analytic's parent company, being pro-active in nutrition labeling, invested heavily in analytical technology to put nutrition information on all of their products. There was a deadline of one year to label more than 6000 different products. The laboratory was built up and throughput enhanced to get this work done on time. A 45 000 square foot laboratory was built and equipped with new instrumentation. Most of the instruments are auto-sampler equipped and all are microprocessor controlled; there are also two Zymark XP robots. These have been custom designed to perform the fatty acid analysis for fatty acid profiles in food products and also for the determination of cholesterol in food products. When the US Government extended the deadlines for compliance with the labeling regulations, Express Analytic had excess capacity within the analytical chemistry laboratory which was made available to the public.

This paper was presented at the 1993 ISLAR, organized by the Zymark Corporation.

This is usually enough to make a scientist think all will be well, but new facilities and new equipment are not enough. With new technology also comes the need for training and development not only on the operational aspects of that equipment, but also on the needs of the people interacting with this new change that has come into the laboratory.

Express Analytic is continuously looking for ways to increase the throughput in the laboratory, and laboratory automation has been the key. In March 1992, two robotic systems were ordered. The first one was delivered in June and was working well by August. In September, the second robot was delivered and by November it was working well. It was not until May 1993 that the first robot was generally recognized as being a good thing to have.

### Problems when introducing robots

Many of the emotions that were encountered upon the introduction of this new technology were feelings such as anxiety or prejudice, fear or blame, hatred, jealousy, doubt, confusion, ridicule, skepticism, apathy and resentment. It is problems like these that the laboratory management must be aware of both as existing and likely to occur, and to deal with them when they do appear.

The primary problem is change or rather the ability (or inability) to deal with change. This is then built upon a lot of the fears associated with change. Change produces stress, and stress can be positive or negative for each individual depending on their behavioral characteristics and the environment within which the stress occurs. In 1914 H. G. Wells in *The World Set Free* had an appropriate quotation regarding change: 'The catastrophe of atomic bombs which shook men out of cities and business and economic relations shook them also out of their old, established habits of thought and out of their lightly held beliefs and prejudices that came down to them from the past'. This view that 'we have always done it this way' is the sort of mentality that is very common and is one that everyone will have to work through when changing environments.

The second problem is ignorance. There are phrases such as: 'They are great on an assembly line, but I don't want one in my lab'. These views have been associated with robots due to the media attention in the past years. When people think robots, they think R2D2 from *Star Wars* or

© Zymark Corporation 1994

Robbie the Robot from *Lost in Space*. This is not what laboratory robotics are, but they certainly have inherited this definition. The negative connotations of the robot come into play here where there are also many associated fears. There is a saying that: 'A man never gets so confused in his thinking that he can't see the other fellow's duty'.

This applies to the laboratory technician who always knows what it is that he is capable of doing and does not want to be replaced by anything. Ignorance plays a very big part in supporting these feelings. It is communication, education and further development of the employees that helps to alleviate these concerns. Also associated with training is a general feeling of inadequacy that may come from the laboratory employees. Phrases like: 'Why should I learn anything new—just get a robot to do it' or 'Great, we just lost the skills that we never had' or 'Now that we have the perfect technician, we can forget all we ever knew about chemistry'. The point here is to address the employee's feelings by looking at the inadequacies of the robot. A manager, speaking to someone who he feels does have these concerns or these feelings of inadequacy, can certainly point out that the robot is deaf, dumb, blind, stupid and 'triphalegic'. Relative to the robot, the employee has quite a few more benefits. The robot is a tool just like an autosampler or any other piece of advanced instrumentation. It is a sample preparation device and nothing more.

Another important reaction is resentment. Phrases like 'You just want me to work more' or 'I wasn't putting out enough work to suit you'. We all have people that come in at 9 and leave at 5, people by whom you can set your watch. Businesses need these people. They are the employees who get the routine work done. The difference between people like this and people who stay until the task is done is the difference between having a job and having a career. The goal of management is to develop career-minded people in every aspect of the business.

Employees whose concerns are in making sure that the job gets done right, making sure that the customer's requirements are met every time, on time, and within budget. Employees who resent the robot are most likely those who need additional training or development in time-management skills or who feel like they are being placed in a spotlight or under pressure to perform. Performance is being required and, in the development of that employee, this could be a very good thing for the business.

A feeling of loss of control is also often encountered—'we could have gotten it done sooner if it weren't for the robot' or 'I couldn't get the analysis done; I've been doing repairs'. In many scientists' minds, a loss of control is also associated with a loss in accuracy, precision or productivity. Given the initial downtime and the learning curve associated with the use of robots, this excuse can be heard more often than we would like. As with any tool, the learning curve can be steep depending on the speed at which the individual learning can assimilate new technology.

A feeling of questionable job security is very often encountered: 'they have a robot now; they don't need me any more'. These are similar to the fears associated with change. In these days where corporations are suffering from employee complacency and feelings of entitlement, the question of job security is often the topic of complaints. Any change in a normal routine tends to generate feelings of threat to people's jobs. In today's business environment, we are in a situation where employee performance is becoming a condition for employment. No business can afford to have people continuing to do today what they have been doing the last two years, yet we all have employees whose tasks have not changed for 20 years.

Any change often puts people at odds with what they have always known. New technology can sneak up on people and initial reactions are very often fear. The fear of not being able to learn or to keep up can cause employee paralysis or often resentment. Resentment in turn, can severely reduce morale and breakup team work and team progress when one member resents anything new or change. Often, we don't have the luxury of removing the person who cannot make the change. This becomes a personnel management issue where the people need to realize that the change is here and the change has to happen. Without change there can be no progress.

The root of all of these problems is fear. A second-grade girl was asked to complete the sentences of some wise sayings that had been read by her teacher. One of the sayings which she was asked to fill in was 'We have nothing to fear but ______'. She filled in 'We have nothing to fear but <u>we're still scared</u>'. It's *the not knowing*. In the laboratory we have a rapidly changing culture, a rapidly changing environment, and any lab that stays on top of the technology is going to have rapidly changing software and hardware. *The not knowing* drives the majority of all worries. This is directly eliminated by efficient and frequent communication with all members of the laboratory community.

A young boy was learning to dive. He was afraid to dive off the 15-foot board. The instructor said: 'you must learn to conquer your fear. What if you were that high up on a sinking ship?' The boy replied: 'I'd wait for the ship to sink another 10 feet, sir'. Our job as managers is fighting this fear. How do we do this? The primary ways of fighting this fear are patience, education, hands-on work, and development of the employees. But, above all else, we must have patience. The importance of training and development cannot be over-emphasized. Any time new technology enters the laboratory, do not undercut yourself or your employees by not providing sufficient or even more than sufficient training for the people. This training builds confidence and familiarity with the equipment that is invaluable. A more educated employee is far more valuable than one who is afraid.

It has been said that the word F.E.A.R. stands for False Evidence Appearing Real. This, also, seems to apply to the case we have with the introduction of the robot. Also note that there is a great difference between training and development. Often the employees know how to run the equipment but developing those skills required to effectively interface not only with the equipment, but also with any of the personnel who need to be involved with the new technology is primary in insuring the success of the operation. Employee morale is basically an issue that we get here. Employees can feel displaced instead of included or ignorant versus trained or confused rather than being informed or ultimately feeling disposable rather than needed. It is the job of a manager to get more work done by directing others than could be done if he were doing the work himself. More has to be done to cover the expensive overhead-eating manager. Employee morale is by far the hardest factor to control. It is hard to build and very easy to crumble. It is like a house made of cards. Often the problems that we encounter in managing technical professionals have new insights that are required for the particular points of view that scientists and other technical professionals have. Too often, it is a scientist who is promoted to manager without having those people-management skills that are necessary to build a cohesive, well functioning team.

Training at Express Analytic has been the key for new people and development has been the key for the experienced employees. Some of the techniques for the introduction of the robot can minimize this by showing the need for robot specialists rather than the need for more basic technicians. This will give the people an opportunity to build their career and to develop their skills to take on the new technology. Position the robot as a fancy autosampler rather than as a synthetic human. It may take some effort depending on your environment to eliminate negative connotations. Offer opportunities to work hands-on with the robot. Familiarity does breed confidence, and this confidence will finally bring pride to the individual who has the opportunity to work with the new equipment. And, finally, emphasize the robot as the tools of the trade. If your laboratory is fortunate to have it, you are still on the cutting edge of technology. This is really a source of pride and can be used as such to help build employee morale.

What do we do if this fails? A number of things can happen. Ultimately, the failure of the team is by far the biggest worry that we have. Given this, it seems to be very worth our time to build on the skills and develop the people within the lab to work with the new technology rather than against it. The result of having a well educated workforce is having an operation that understands the effect that their work has on the business as well as those around them. Invest in employee communication and training. It is well worth the time, money and effort.

